# MeCP2-mediated epigenetic regulation in senescent endothelial progenitor cells

**DOI:** 10.1186/s13287-018-0828-y

**Published:** 2018-04-03

**Authors:** Chunli Wang, Fei Wang, Zhen Li, Qing Cao, Liya Huang, Shuyan Chen

**Affiliations:** Department of Geriatrics, Xinhua Hospital Affiliated to Shanghai Jiao Tong University School of Medicine, Shanghai, China

**Keywords:** Endothelial progenitor cells, Senescence, MeCP2, SIRT1, Epigenetic

## Abstract

**Background:**

Cellular aging may be associated with epigenetics. Methyl-CpG-binding protein 2 (MeCP2) and sirtuin 1 (SIRT1) are two important epigenetic factors. Our former work demonstrated that MeCP2 expression increased and SIRT1 expression decreased in senescent endothelial progenitor cells (EPCs). This article aims to reveal the epigenetic regulation caused by MeCP2 in EPCs and discuss its mechanism.

**Methods:**

Tube formation assay and cell apoptosis detection were used to evaluate the function of senescent EPCs induced by MeCP2 overexpression. Western blot analysis was used to testify the relative protein expression changed by MeCP2. Bisulfite sequencing methylation assay and chromatin immunoprecipitation assay were used to assess the degree of methylation and the relation of MeCP2 and SIRT1.

**Results:**

MeCP2 reduced angiogenesis of senescent EPCs, promoted apoptosis, and caused senescent EPC dysfunction through SIRT1 promoter hypermethylation and histone modification.

**Conclusions:**

MeCP2 mediated senescent EPC dysfunction through epigenetic regulation.

## Background

Aging can be modified by environmental and genetic factors [1], it is a gradual loss of homeostatic mechanism that maintains the structure and function of adult tissues [2]. Cellular senescence promotes aging [3, 4]. Senescent cells show functional insufficiency or defect. Our previous work has demonstrated that senescent endothelial progenitor cells (EPCs) had decreased cell proliferation activity, migration capacity, and tube formation ability and increased susceptibility to apoptosis.

Unbalanced epigenetic regulation is considered to contribute to the progression of aging [5, 6]. Two major epigenetic mechanisms influence chromatin structure: histone modifications and DNA methylation [7]. Histone modifications modulate the degree of compaction of nucleosomes, thereby affecting chromatin accessibility to various factors, particularly transcriptional regulators [8–10]. It has been demonstrated that H3K9 methylation is associated with transcription repression [11]. DNA methylation consists of adding a methyl group to cytosines (particularly at the C5 position of cytosine in cytosine–guanine dinucleotide sequences (CpG)) by DNA methyltransferases (DNMT), creating 5-methylcytosine (5-mC) [12, 13]. 5-mC is bound by methyl-binding proteins (such as MeCP2), which recruit other protein partners, including HDAC and mSin3A, forming corepressor complexes [14, 15].

MeCP2 is an important member of the methyl-CpG-binding protein family. MeCP2 mutation was first identified as the primary cause of the neurological Rett syndrome (RTT) by Amir et al. [16]. Our former work found senescent EPCs had elevated MeCP2 expression and decreased SIRT1 expression [17]. Now we will illuminate the epigenetic regulation of MeCP2/SIRT1 in senescent EPCs.

## Methods

### EPC isolation and culture

This work was approved by the Ethics Committee of Xinhua Hospital Affiliated to Shanghai Jiao Tong University, Shanghai, China, and consent from donors was received. Human cord blood mononuclear cells (MNCs) isolated by density gradient centrifugation with Histopaque-1077 (Sigma) were suspended in complete EGM-2 medium (Lonza Clonetics) and then seeded on fibronectin (Gibco) precoated 24-well plates. Approximately 9 × 10^6^ cells were collected from 20 ml of cord blood, and 1.5 × 10^6^ cells were plated per well. Medium was changed every 3 days until the first passage. For identification of an EPC, cells were stained with antibodies against endothelial markers CD34 and VEGFR-2 as well as stem/progenitor marker CD133 by fluorescence microscopy, and by dual staining for acetylated low-density lipoprotein (Dil-ac-LDL; Molecular Probes) uptake and UEA-1 (Sigma) binding.

### Immunofluorescence

About 1 × 10^5^ cells were fixed with 4% paraformaldehyde for 20 min. They were then permeabilized with 0.3% Triton X-100. After that, cells were incubated consecutively with primary antibodies (CD34, CD133, VEGFR-2, MeCP2, and SIRT1) overnight at 4 °C and secondary antibodies (ALEXA-488 conjugate or CY3 conjugate) for 2 h at room temperature. For the uptake of Dil-Ac-LDL and binding of FITC-UEA-1 assay, EPCs were incubated with Dil-Ac-LDL overnight at 37 °C. They were then fixed with 4% paraformaldehyde for 20 min. After that, the cells were incubated with FITC-UEA-1 for 1 h at 37 °C. After nucleus staining with DAPI dye, cells were examined under a fluorescent microscope.

### Flow cytometry analysis for cell apoptosis

Cell apoptosis was detected by dual staining with PE Annexin V and 7-AAD (BD, Biosciences). The medium used for cell apoptosis assay was EGM-2 complete medium. About 1 × 10^5^ cells in 1 ml medium were seeded per well in 24-well plates in triplicate with GFP, Ad-MeCP2, or Ad-sh-MeCP2. Adherent and nonadherent cells were harvested and resuspended in Annexin-binding buffer, stained with PE Annexin V and 7-AAD for 15 min at room temperature protected from light, and then analyzed with flow cytometry (BD FACSCanto II) as soon as possible.

### TUNEL assay

The slides of cells were transfected with Ad-GFP, Ad-MeCP2, and Ad-sh-MeCP2. After washing with PBS, cells were fixed with 4% paraformaldehyde for 15 min, washed with PBS three times, and added to 500 μl 0.1% Triton-X100 for 10 min at room temperature. After washing with PBS three times, the cells were added to 50 μl TUNEL (Roche) at 37 °C for 1 h. Washed with PBS, the cells were added to DAPI at room temperature for 5 min, and then observed with a fluorescence microscope.

### Western blot analysis

Cell proteins were extracted by RIPA. The protein concentration was measured with the BCA method. Approximately 30 μg of protein from each sample was loaded on 8% SDS–PAGE gels and run at 80 V constant voltage. A constant current of 300 mA was used for transblotting. Blots were probed with rabbit anti-MeCP2 antibodies (1:1000; Abcam) and anti-SIRT1 antibodies (1:2000; Abcam) overnight at 4 °C. After washing three times, blots were then incubated with goat anti-rabbit secondary antibody (1:1000) at room temperature for 2 h. Chemiluminescence was then used to visualize protein bands. The housekeeping gene anti-β-actin antibody (1:1000; Abcam) was used as control.

### Matrigel angiogenesis assay

μ-Slide Angiogenesis dishes (ibidi, Germany) were used according to the manufacturer’s instructions. Matrigel (10 μl; BD Biosciences) was coated on each μ-slide angiogenesis well and incubated at 37 °C for 30 min, and EPCs were seeded on the Matrigel-coated μ-slide angiogenesis well plate at a density of 1 × 10^4^ cells per well. Complete EGM-2 (50 μl) was used as the medium for the assay. Images were captured 24 h after seeding, and total loops were measured.

### Adenovirus transfection

Adenoviral vectors containing green fluorescent protein (GFP) and harboring wild-type MECP2 (Ad-MeCP2) and short hairpin RNA MeCP2 (Ad-sh-MeCP2) were purchased from Han Heng (Shanghai, China). Adenoviral vectors carrying only GFP (Ad-GFP) were used as the controls. EPCs were transfected with Ad-GFP, Ad-MeCP2, and Ad-sh-MeCP2 at a multiplicity of infection (MOI) of 80 and incubated for 4 h. Afterward, the media were exchanged for fresh EGM-2, and the transfected EPCs were cultured for an additional 44 h. The effects of Ad-MeCP2 and Ad-sh-MeCP2 on the expression of MeCP2 were detected by western blot and RT-PCR analysis in our former study.

### Bisulfite sequencing-based SIRT1 promoter methylation assay

For analysis of SIRT1 promoter methylation, genomic DNA was isolated using the Genomic DNA Mini Preparation Kit with Spin Column (Beyotime) and bisulfite converted with the EpiTect Kit (Qiagen). Primers of a CpG island within 2 kb upstream of the initiation codon of SIRT1 were designed with MethPrimer (http://www.urogene.org/cgi-bin/methprimer/methprimer.cgi). Sequences of primers were: forward, 5′-AAGGTTAAGGTAGGTTAGGTGT-3′; and reverse, 5′-CCAACTACCTCTCTAACCCT-3′. PCR products were subcloned into pCR2.1 vector with the TopoTA cloning Kit (Invitrogen). Six colonies were picked, plasmid DNA was purified, and DNA was sequenced. The degree of methylated DNA was analyzed with QUMA (http://quma.cdb.riken.jp/top/index.html).

### Chromatin immunoprecipitation

To study a potential association of MeCP2 with the SIRT1 promoter, chromatin-bound MeCP2 was immunoprecipitated using a MeCP2 antibody (ab2828; Abcam) as per the manufacturer’s instructions followed by quantitative PCR for the SIRT1 promoter. As MeCP2 recruits histone methytransferase activity directed against lysine 9 of histone 3 which is linked to gene silencing, a repressive epigenetic chromatin mark, Histone 3 Lysine 9 dimethylation (H3K9me2), was used on nucleosomes within the SIRT1 promoter. The SIRT1 promoter was immunoprecipitated using H3K9me2 antibody (ab32521; Abcam). Primer sequences used to amplify the SIRT1 promoter containing CpG islands were as follows: forward, 5′-GAGACGGAGTTTCGCTCTTG-3′; and reverse, 5′-CCTGAAGTCGGAAGTTCGAG-3′.

### Statistical analysis

Results are expressed as the mean ± SE. Comparisons between groups were analyzed by paired-sample *t* test when appropriate. A probability value of *P* ≤ 0.05 was considered statistically significant. All analyses were performed with SPSS 13.0 software.

## Results

### Culture and identification of EPCs

**Fig. 1:**
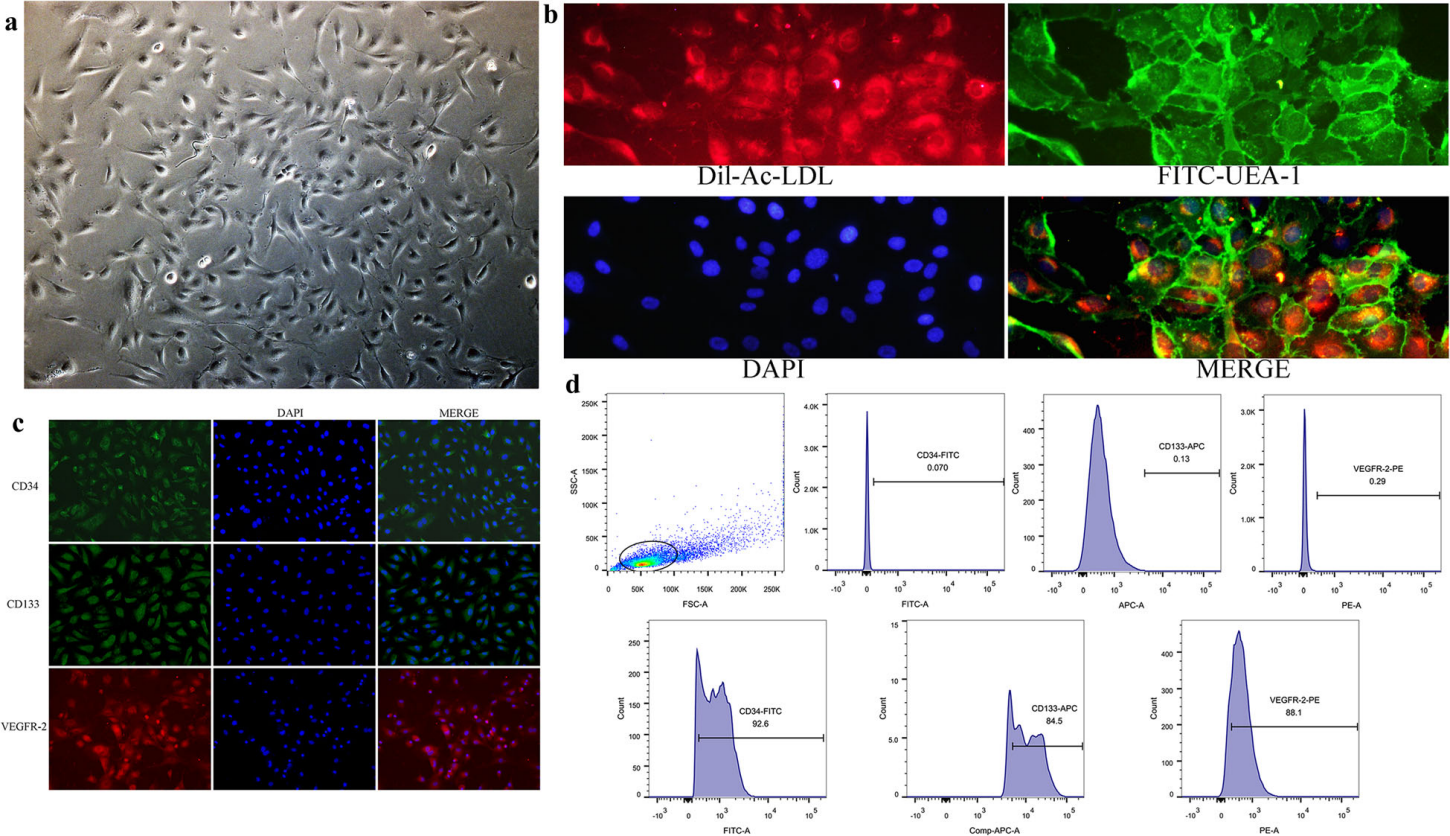
Cultivation and identification of EPCs derived from umbilical cord blood. **a** EPCs exhibited a cobblestone-like cell monolayer at 14 days after seeding (×100). **b** Uptake of Dil-Ac-LDL and binding of FITC-UEA-1 observed with a fluorescence microscope (×400). **c** Typical fluorescence images of expression of CD34, CD133, and VEGFR-2 in EPCs (×200). **d** Representative FSC/SSC plot and expression of EPC markers (CD34, CD133, and VEGFR-2) analyzed by flow cytometry. DAPI 4',6-diamidino-2-phenylindole, VEGFR vascular endothelial growth factor receptor, FITC fluorescein isothiocyanate, APC allophycocyanin, PE phycoerythrin

Under the present culture conditions, the adherent cells from human cord blood MNCs appeared in colonies and showed a cobblestone morphology after 2 weeks of culture (Fig. 1a), and were double-positive stained for Dil-Ac-LDL and FITC-UEA-I (Fig. 1b). EPCs were confirmed by examining the expression of endothelial cell surface antigen VEGFR-2 and progenitor cell surface antigens CD34 and CD133, as shown in Fig. 1c. Flow cytometry analysis revealed that the positive expression rate of CD34 was 92.7 ± 2.55%, and was 84.33 ± 3.85% for CD133 as well as 87.26 ± 2.84% for VEGFR-2 (Fig. 1d). All of these are considered important characteristics of EPCs.

### Location of MeCP2 and SIRT1 in EPCs

**Fig. 2:**
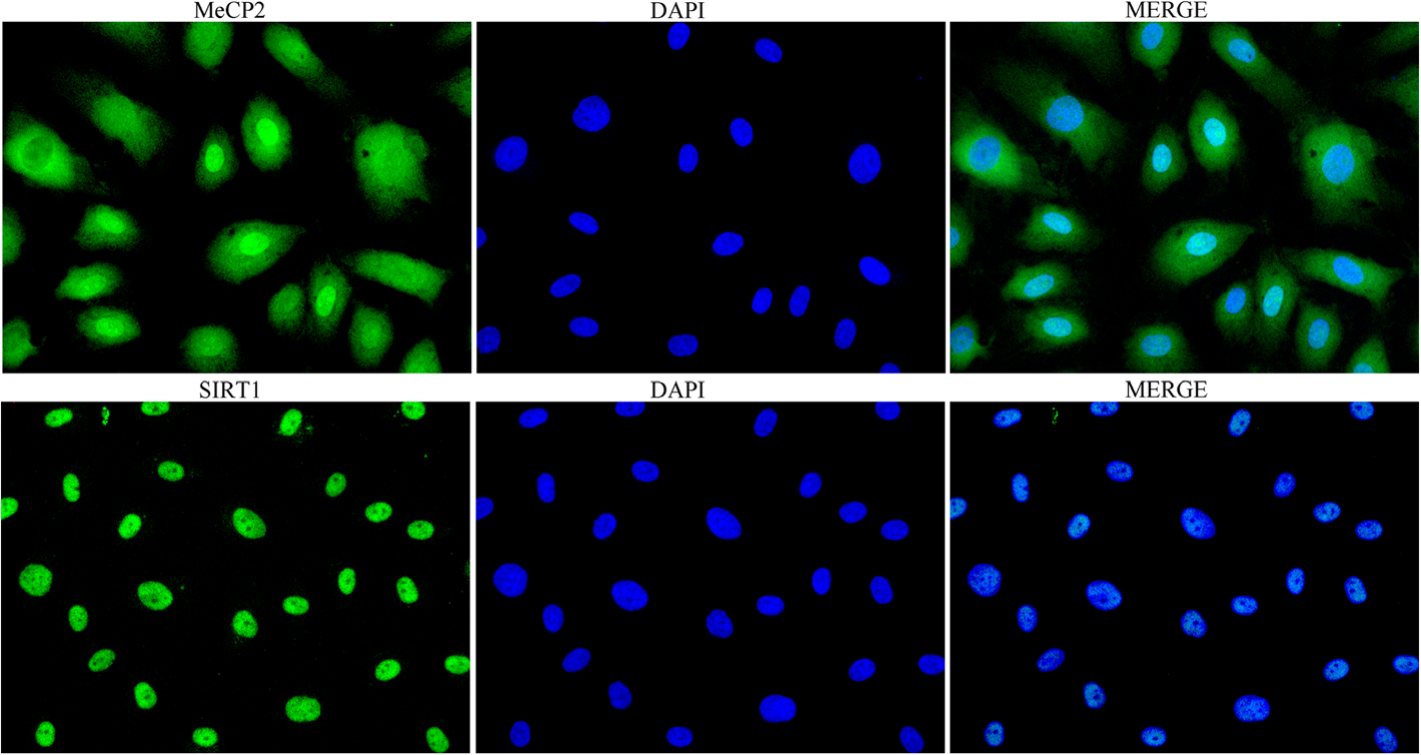
Expression of MeCP2 and SIRT1 in EPCs. Immunofluorescence shows SIRT1 localized in the nucleus and MeCP2 localized in the nucleus and cytoplasm (×200). MeCP2 methyl-CpG-binding protein 2, DAPI 4',6-diamidino-2-phenylindole, SIRT1 sirtuin 1

Figure 2 shows that MeCP2 located in the cell nucleus and cytoplasm of EPCs, while SIRT1 only located in the cell nucleus.

### MeCP2 inhibited SIRT1 in senescent EPCs

We found SIRT1 expression was negatively regulated by MeCP2 in our previous work. EPCs were transfected with Ad-GFP, Ad-sh-MeCP2, and Ad-MeCP2. The confirmation of transfection efficiency was also seen in our former work [17].

**Fig. 3:**
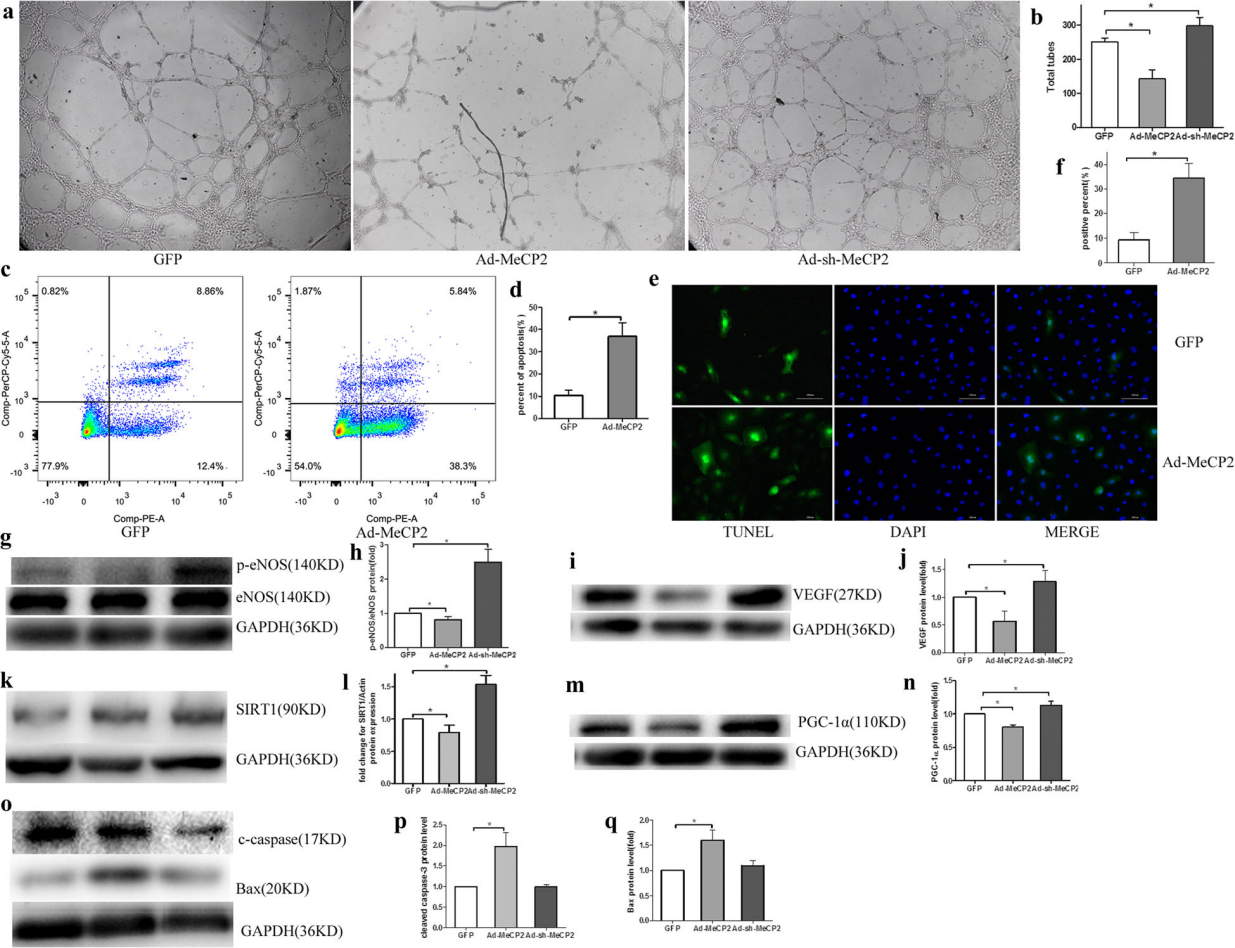
MeCP2 mediated EPC dysfunction. **a**, **b** MeCP2 induced EPC angiogenesis reduction (×40, total tubes used to evaluate tube formation ability). **c**, **d** Flow cytometry shows MeCP2 overexpression promoted cell apoptosis (percentage of apoptotic cells with PE Annexin V-positive expression used to assess apoptosis). **e**, **f**, TUNEL fluorescence staining shows MeCP2 increased the number of apoptosis cells. **g**–**j** MeCP2 reduced angiogenesis relative protein levels, p-eNOS/eNOS (**g**, **h**) and VEGF (**i**, **j**). **k, l** Protein levels of SIRT1 with MeCP2 overexpression or silence. **m**, **n** Protein levels of PGC-1α with MeCP2 overexpression or knockout. **o**–**q** MeCP2 regulated apoptosis relative proteins. Data reported as mean ± SD, *n* = 3. *P < 0.05 vs GFP. GFP green fluorescent protein, MeCP2 methyl-CpG-binding protein 2, TUNEL terminal deoxynucleotidyl transferase dUTP nick end labeling, DAPI 4',6-diamidino-2-phenylindole, eNOS endothelial nitric oxide synthase, p-eNOS phospohorylated eNOS, SIRT1 sirtuin 1, GAPDH glyceraldehyde 3-phosphate dehydrogenase

The tube formation assay results showed that MeCP2 repressed the angiogenesis activities of EPCs (Fig. 3a, b), Flow cytometry analysis and TUNEL assay confirmed that MeCP2 promoted EPC apoptosis (Fig. 3c–f).

Western blot analysis results displayed endothelial nitric oxide synthase (eNOS), phospho-eNOS (p-eNOS), and angiogenesis activities of VEGF expression were decreased by MeCP2 overexpression (Fig. 3g–j). SIRT1 was downregulated by MeCP2 overexpression (Fig. 3k, l). Western blot analysis also showed a positive correlation between MeCP2 and Bax, and cleaved caspase-3 expression (Fig. 3o–q), while our former work found SIRT1 had a negative correlation with the expression of Bax and cleaved caspase-3 [18]. PGC-1α, which can be activated by SIRT1, was repressed by MeCP2 (Fig. 3m, n).

### MeCP2 led to SIRT1 promoter hypermethylation

**Fig. 4:**
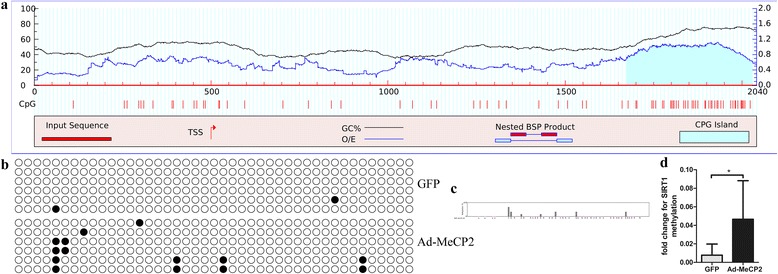
Methylation analysis of SIRT1 CpG islands. **a** One CpG island in SIRT1 promoter region. **b**, **c**, **d** Bisulfite promoter analysis for SIRT1 methylation after viral MeCP2 overexpression (black presents methylation location). Data reported as mean ± SD, *n* = 3. *P < 0.05 vs GFP. CpG cytosine-guanine dinucleotide sequences, TSS transcriptional start site, GFP green fluorescent protein, MeCP2 methyl-CpG-binding protein 2, SIRT1 sirtuin 1

Through DNA sequence analysis, we found SIRT1 possessed one CpG island in its promoter region within 2000 bp upstream of the transcriptional start site (Fig. 4a). BSP showed that the methylation status of the SIRT1 promoter was significantly increased by MeCP2 overexpression (Fig. 4b–d).

### MeCP2, H3K9me2 binds to SIRT1 promoter

**Fig. 5:**
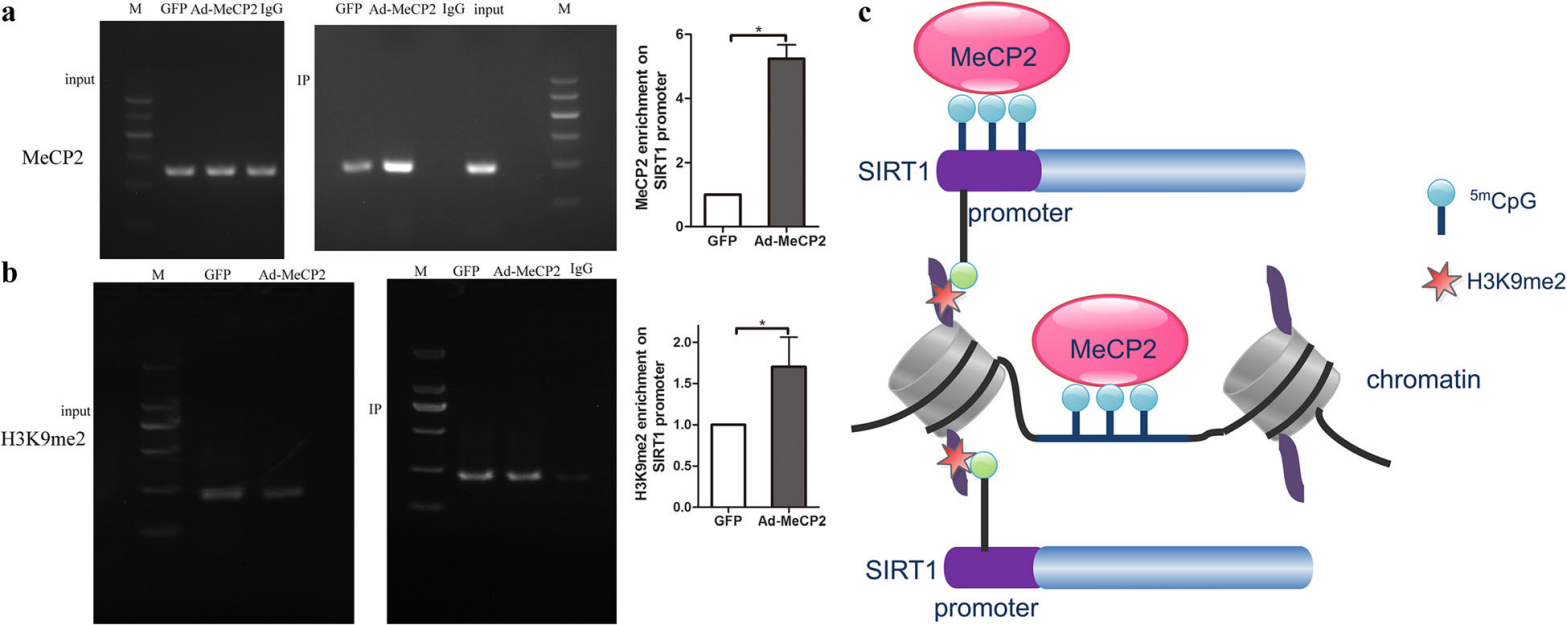
Mechanism of MeCP2-inhibited SIRT1 transcription. **a** ChIP against MeCP2 shows more MeCP2 enrichment on SIRT1 promoter in MeCP2-overexpressed EPCs. **b** ChIP against H3K9me2 shows more H3K9me2 enrichment on SIRT1 promoter in MeCP2-overexpressed EPCs than control. **c** Proposed mechanism of senescent EPC dysfunction. *n* = 3. *P < 0.05 vs GFP. GFP green fluorescent protein, MeCP2 methyl-CpG-binding protein 2, H3K9me2 Histone 3 Lysine 9 dimethylation, SIRT1 sirtuin 1, CpG cytosine-guanine dinucleotide sequences

Chromatin immunoprecipitation (ChIP) assays showed enrichment of MeCP2 on SIRT1 promoter (Fig. 5a). MeCP2 mediated epigenetic gene repression not only through DNA methylation, but also through repressive H3K9me2 marks. We performed ChIP using an anti-H3K9me2 antibody and primers specific for SIRT1 promoter. MeCP2 enhanced the presence of H3K9me2 in nucleosomes of the SIRT1 promoter (Fig. 5b). The mechanism of MeCP2 mediating the regulation of EPC dysfunction is shown in Fig. 5c.

## Discussion

Umbilical cord blood-derived EPCs display an endothelial phenotype associated with progenitor cell features such as clonal growth and high proliferation [19, 20]. Gradual loss of cellular functions including the ability to proliferate with an increase in the number of cell divisions during cell population development was called replicative aging or cellular senescence [21]. During aging, the cell undergoes substantial changes in functional activity, morphology, and proliferative potential [22, 23]. The number of EPCs decreases with aging, along with their ability for proliferation, migration, and angiogenesis [24, 25], as we found formerly in senescent EPCs.

MeCP2 is a chromosomal protein that is able to bind methylated DNA via a methyl-binding domain (MBD) [26]. It was reported that MeCP2 located to the cortex nucleus and concentrated at heterochromatic foci in transgenic mice [27]. Here we found that MeCP2 appeared as a nucleus and cytoplasm protein. SIRT1 is predominantly localized in the nucleus [28], and we found the same location. SIRT1 promotes vascular relaxation by activating endothelial nitric oxide synthase (eNOS) [28], and promotes angiogenesis by increasing VEGF expression [29]. Our previous work showed that SIRT1 can improve EPC function. In this study we demonstrate that MeCP2 leads to reduced angiogenesis marker - active eNOS and VEGF, which both could be activated by SIRT1. We also find MeCP2 can induce EPC apoptosis, which can be reversed by SIRT1. Besides, we find MeCP2 leads to reduced PGC-1α, which can be activated by SIRT1 [30, 31]. So it is concluded that MeCP2 can repress SIRT1.

Aging is at least in part, if not largely, a manifestation of epigenetic changes [32]. MeCP2 is an important epigenetic factor. It binds to methylated DNA [33], unmethylated DNA [34, 35], or hemimethylated DNA [36] to induce DNA methylation, resulting in alterations in gene expression pattern [37]. DNA methylation, a major epigenetic factor, is essential for silencing retroviral elements, regulating tissue-specific gene expression, genomic imprinting, and X-chromosome inactivation, and the majority of methylation occurs within CpG sites. CpG islands are likely to enhance binding to transcriptional start sites. Methylation of CpG islands can impair transcription factor binding, recruit repressive methyl-binding proteins, and stably silence gene expression [38]. With age, mammalian cells undergo global DNA hypomethylation and local DNA hypermethylation (especially CpG cites) [39]. We find that SIRT1 promoter CpG hypermethylated with MeCP2 overexpression and MeCP2 enrichment on the SIRT1 promoter. In addition, we also find H3K9me2 enriched in the SIRT1 promoter with MeCP2 overexpression.

## Conclusions

Manifestations show the causes of aging may be largely epigenetic [40]. This article finds that MeCP2 inhibits SIRT1 to induce senescent EPC dysfunction in two ways in senescent EPCs: one is DNA hypermethylation, and the other is histone hypermethylation.

Our study also has several limitations. The ageing process is slow and complicated, and many regulators and signaling pathways are involved rather than a single factor. We identified MeCP2 and SIRT1, but there may be other regulators. MeCP2 may inhibit SIRT1 through other factors.

## Abbreviations

ChIP: Chromatin immunoprecipitation
CpG: Cytosine–guanine dinucleotide sequences
DNMT: DNA methyltransferases
eNOS: Endothelial nitric oxide synthase
EPC: Endothelial progenitor cell
H3K9me2: Histone 3 Lysine 9 dimethylation
MBD: methyl-binding domain
MeCP2: Methyl-CpG-binding protein 2
SIRT1: Sirtuin 1
